# fNIRS en la Evaluación Emocional y la Corteza Prefrontal Dorsolateral: Una Revisión Sistemática

**DOI:** 10.31083/RN44275

**Published:** 2025-11-30

**Authors:** Ivonne Carpio-Toro, Edwin Alberto Maxi Maxi, Gerardo Beltrán Serrano, Andrés Ramírez, Joan Deus Yela

**Affiliations:** ^1^Departamento de Psicología Clínica y de la Salud, Universidad Autónoma de Barcelona, 08193 Barcelona, España; ^2^Universidad Católica de Cuenca, 010107 Cuenca, República del Ecuador; ^3^Programa de Postgrado en Ciencias Médicas, Facultad de Medicina, Universidade Federal do Rio Grande do Sul (UFRGS), 90035-003 Porto Alegre, Brasil; ^4^Laboratorio de Dolor y Neuromodulación, Hospital de Clínicas de Porto Alegre (HCPA), 90035-003 Porto Alegre, Brasil

**Keywords:** espectroscopia funcional de infrarrojo cercano, fNIRS, NIRS, corteza prefrontal, emoción, functional near infrared spectroscopy, fNIRS, NIRS, prefrontal cortex, emotion

## Abstract

**Antecedentes:**

El presente estudio aborda una revisión sistemática sobre el estudio de tareas emocionales mediante el uso de la espectroscopía de infrarrojo cercano (fNIRS) en la corteza prefrontal (CPF), remarcando la comprensión de los procesos subyacentes a la neurocognición y emoción en diversos contextos, mediante la medida de oxigenación en la CPF como indicador de activación cerebral.

**Objetivo:**

Revisión detallada de la investigación actual sobre el uso de la fNIRS en la medición de la actividad de la CPF dorsolateral (CPFDL) en tareas que evalúen aspectos emocionales en adultos.

**Metodología:**

Búsqueda exhaustiva de estudios en bases de datos PubMEd, Scopus, Web of Science, utilizando metodología PRISMA, el análisis de sesgo y criterios de inclusión específicos. La selección de estudios se basó en la calidad metodológica y relevancia temática, seguida de la extracción y análisis de datos.

**Resultados:**

La población estudiada incluye adultos sanos y pacientes con trastornos mentales. Los instrumentos y configuraciones técnicas de fNIRS fueron diversos y los experimentos emocionales abarcaron varias tareas, revelando patrones de activación cerebral en las tareas que involucran un procesamiento emocional; se observa una alteración de CPFDL izquierda lo que puede ser indicador de patología y una activación CPFDL derecha ante estímulos agradables lo cual abre una puerta para futuras investigaciones e intervenciones sobre lateralidad cerebral.

**Conclusión:**

Se resalto la complejidad de los procesos neurocognitivos y emocionales, señalando a la CPFDL como un área importante con implicaciones en psicología, neurociencia y salud mental. Se enfatiza la necesidad de considerar diversos factores contextuales y metodológicos en su investigación, como el tiempo de exposición a las tareas que permita una adecuada recepción de la señal.

## Abstract (en)

**Background:**

This study presents a systematic review on the use of functional near-infrared spectroscopy (fNIRS) in emotional tasks involving the prefrontal cortex (PFC), emphasizing the understanding of neurocognitive and emotional processes in various contexts through the measurement of oxygenation in the PFC as an indicator of brain activation.

**Objective:**

To provide a detailed review of current research on the application of fNIRS to assess activity in the dorsolateral prefrontal cortex (DLPFC) during emotional processing tasks in adults.

**Methods:**

A comprehensive literature search was conducted in PubMed, Scopus, and Web of Science databases, following PRISMA guidelines, with specific inclusion criteria and bias analysis. Study selection was based on methodological quality and thematic relevance, followed by data extraction and analysis.

**Results:**

The studied population includes healthy adults, and patients with mental disorders. The instruments and technical settings of fNIRS were diverse, and the emotional experiments involved various tasks, revealing patterns of brain activation in tasks that involve emotional processing; with altered left DLPFC activation can be observed in clinical populations, suggesting potential biomarkers of pathology. In contrast, right DLPFC activation in response to pleasant stimuli points to possible implications for future research and interventions related to cerebral laterality.

**Conclusion:**

The review highlights the complexity of neurocognitive and emotional processes, underscoring the relevance of the DLPFC in psychology, neuroscience, and mental health. It also emphasizes the need to consider various contextual and methodological factors in future studies, such as adequate exposure time to tasks for optimal signal acquisition.

## 1. Introducción

En las últimas décadas, la espectroscopia funcional de infrarrojo cercano (fNIRS) ha emergido como una herramienta crucial en neurociencia cognitiva debido a su capacidad para medir la activación cortical de manera no invasiva, portátil y segura, superando limitaciones de técnicas como resonancia magnética funcional (RMf) y el electroencefalograma (EEG) en ciertos contextos [1]. El fNIRS utiliza longitudes de onda de luz específicas dentro de la ventana óptica; una vez que los fotones se introducen en el tejido, se dispersan por los límites intracelulares y extracelulares de las diferentes capas (piel, cráneo, líquido cefalorraquídeo, cerebro, entre otras). La luz es absorbida principalmente por la oxihemoglobina (HbO) y desoxihemoglobina (HbR), lo que permite inferir la actividad neuronal, ya que las áreas cerebrales activas requieren mayor consumo de oxígeno [2, 3].

Este dispositivo óptico permite monitorizar la absorción de la luz en el espectro de infrarrojo cercano (750 nm y 950 nm) a través del cráneo intacto; lo que permite determinar los cambios de concentración del oxígeno en sangre a partir de mediciones de luz difusa dispersa, con una profundidad de hasta, aproximadamente, 3 centímetros [4].

Recientemente, investigaciones sobre fNIRS, enfatizan la capacidad adecuada para medir hemoglobina y su uso en comparación con EEG y RMf, enfatizando tendencias y prioridades en la investigación futura [5]. Esta técnica de neuroimagen se destaca por ser una alternativa cómoda y menos restrictiva en comparación con la RMf, ya que ofrece múltiples ventajas como las siguientes: no es invasiva, es portátil, de costo relativamente bajo, fácil de manejar, que permite mediciones continuas y repetidas en los participantes en entornos naturales o en consultorio [4, 6].

De esta manera, el fNIRS no emplea campos magnético ni gradientes de alta intensidad, lo que hace seguro para una amplia gama de participantes, incluidos niños o personas con implantes metálicos [7]. A diferencia de EEG, el fNIRS permite utilizar la técnica en contextos más abiertos, como la evaluación durante actividades físicas, donde es fundamental que los participantes se mantengan en movimiento [8]. Una última ventaja notable es que los sujetos pueden ser probados simultáneamente y mientras interactúan, por lo que es una herramienta ideal para experimentos de neurociencia social [9, 10] (véase en la Tabla 1).

**Tabla 1.** **Comparativa de fNIRS, RMf y EEG**.

Característica	fNIRS	RMf	EEG
Variable medida	Cambios en la concentración de hemoglobina oxigenada (HbO) y desoxigenada (HbR) como indicador del flujo sanguíneo regional.	Imagen detallada de la actividad cerebral, en base a la concentración de HbO en fase venosa (efecto BOLD), mediante campos magnéticos y ondas de radio.	Actividad eléctrica del cerebro a través de cambios en el campo eléctrico generado por las neuronas.
Principio	Espectroscopia de infrarrojo cercano: mide la absorción de luz en tejidos biológicos.	Campos magnéticos y señales de radiofrecuencia para reconstruir imágenes cerebrales en 2D o 3D.	Potenciales eléctricos, recogidos en la superficie del cuero cabelludo.
Resolución espacial	Baja (~1–3 cm) limitada a la corteza superficial.	Alta (~1–2 mm) para toda la estructura cerebral.	Baja (~1–2 cm) debido a la dispersión del campo eléctrico.
Resolución temporal	Moderada (~100 ms), adecuada para eventos neurocognitivos.	Baja (~1–2 s) debido al tiempo necesario para adquirir múltiples imágenes.	Excelente (~1 ms), captando actividad en tiempo real.
Comodidad del usuario	Alta: no invasiva, portátil, adecuada para entornos ecológicos.	Moderada: no invasiva pero requiere inmovilización y entorno controlado.	Alta: no invasiva, pero depende de la configuración de los electrodos.
Costo	Moderado, depende del número de canales y configuraciones técnicas.	Alto debido al equipo especializado y mantenimiento.	Moderado, con sistemas asequibles disponibles.
Aplicaciones	Estudios de activación cerebral, neuropsicología neuroergonomía, neurofeedback, neuropsiquiatría.	Estudios de activación cerebral en neurociencias y comprender sus bases neurobiológicas, localización de funciones críticas antes de cirugías cerebrales.	Diagnóstico de epilepsia, trastornos del sueño, encefalitis y algunas enfermedades neurodegenerativas.

*Nota. *fNIRS, Espectroscopia funcional de infrarrojo cercano; RMf, Resonancia Magnética functional; EEG, Electroencefalograma.

Existen diferentes modelos de fNIRS dependiendo de las marcas que los distribuyen. Sin embargo, todos ellos están formados por emisores y receptores de luz infrarroja cercana dispuestos en un gorro o cinta, dependiendo del equipo utilizado. La ubicación de los sensores están en relación al sistema internacional 10–20 para asegurar una colocación estandarizada sobre las regiones cerebrales de interés [3, 11].

Consecuentemente estudios recientes han demostrado la utilidad de esta técnica para explorar la compleja relación entre la actividad cerebral, los procesos neurocognitivos y aspectos afectivos [6, 12, 13], lo que proporciona información sobre la actividad funcional cerebral al instante y de forma interactiva [14, 15]. Por ejemplo, Manelis y colaboradores (2019) [16], investigaron las diferencias en la actividad de la corteza prefrontal (CPF) en individuos deprimidos versus controles sanos durante el reconocimiento de expresiones emocionales. Se encontró que los individuos con depresión tenían dificultades para reconocer expresiones neutras, lo que se correlacionó con una menor activación cortical, reflejando afectaciones en la regulación emocional y el control neurocognitivo [17]. Este estudio, uno de los primeros en explorar el reconocimiento de expresiones faciales emocionales en individuos con un trastorno afectivo tipo depresivo y destaca su aporte al análisis metodológico del uso de esta técnica de neuroimagen. Además, pone en cuestión la práctica común de utilizar estímulos neutrales como referencia para comparar emociones. Consecuentemente esta metodología podría no ser adecuada en todos los casos, especialmente en individuos con trastornos psicopatológicos, ya que el procesamiento de rostros neutros puede variar significativamente en estos grupos. Estas diferencias podrían sesgar las comparaciones y afectar los resultados, como se evidenció mediante la aplicación de esta herramienta.

La corteza prefrontal dorsolateral (CPFDL) es crítica para funciones como la regulación emocional, el control inhibitorio, la toma de decisiones que permite adaptarnos y mantener relaciones sociales saludables [18]. También es importante para la atención y la memoria de trabajo, que mantiene la información temporalmente activa para su uso inmediato, lo que es crucial para tareas como comprender instrucciones, resolver problemas y razonar [17], lo que la convierte en un objetivo clave para estudios de neuroimagen centrados en la emoción.

Sin embargo, a pesar de la importancia de estas funciones, aún se requiere más investigación para comprender completamente cómo la actividad de la CPF se relaciona con las emociones en diferentes contextos y tareas [13], señalando al fNIRS como una herramienta útil para estudiar la actividad cerebral [3, 6, 18]. Aunque el fNIRS ha demostrado ser una herramienta eficaz para investigar la actividad cerebral en procesos emocionales y neurocognitivos, la medición directa de las emociones sigue siendo un desafío significativo para la psicología y las neurociencias [3, 6]. Dado que la CPF juega un papel crucial en la regulación emocional, es fundamental seguir investigando cómo estas interacciones se reflejan en biomarcadores, como la oxigenación cerebral [3, 9, 19].

Por lo tanto, el objetivo de esta revisión sistemática es proporcionar una visión detallada de la investigación actual sobre el uso de fNIRS para evaluar la actividad CPFDL durante la ejecución de tareas emocionales en población adulta.

## 2. Metodología 

### 2.1 Diseño

Se realizó una revisión sistemática basada en los criterios establecidos por la declaración Preferred Reporting Items for Systematic Reviews and Meta-Analyses (PRISMA) [20]. La lista de verificación PRISMA 2020 se proporciona en el **Material Suplementario–PRISMA 2020 checklist**.

### 2.2 Criterios de Selección

Se incluyeron estudios que cumplieran con los siguientes criterios: (1) artículos publicados entre enero de 2013 y enero de 2024 en revistas científicas con revisión por pares; (2) textos disponibles en inglés o español; (3) uso de fNIRS para la evaluación de la CPFDL; (4) evaluación de aspectos emocionales en población adulta (18–65 años); (5) pertenencia a las áreas de psiquiatría, medicina, neuropsicología, psicología o neurociencia.

Se excluyeron: (1) estudios con muestras no humanas; (2) investigaciones en niños o adultos mayores de 65 años; (3) estudios que no se enfoquen en la CPF o CPFDL; (4) investigaciones que no reporten medidas de HbO y desoxigenada (HbR); (5) estudios que utilicen técnicas distintas a fNIRS; (6) literatura gris, como tesis, capítulos de libros, manuales, actas de congresos o estudios de caso único.

### 2.3 Fuentes de Información

La búsqueda se realizó en tres bases de datos: PubMed, Web of Science (WOS) y Scopus, seleccionadas por su cobertura científica y rigurosidad editorial en ciencias de la salud y ciencias sociales. El acceso se efectuó a través de las bibliotecas de la Universidad Autónoma de Barcelona (España) y la Universidad Católica de Cuenca (Ecuador).

### 2.4 Estrategia de Búsqueda

Se emplearon operadores booleanos y descriptores controlados (DeCS/MeSH). Los términos clave fueron: “Dorsolateral Prefrontal Cortex”, “DLPFC”, “fNIRS” y “Functional Near-Infrared Spectroscopy”, combinados con “adult” y “emotions”. Se aplicaron filtros por fecha (últimos 10 años), idioma (inglés y español) y áreas temáticas afines (psicología, medicina, neurociencia). Los detalles se presentan en la Tabla 2.

**Tabla 2.** **Ecuación de búsqueda por base de datos**.

Base de datos	Ecuación de búsqueda
PubMed	(((Dorsolateral Prefrontal Cortex OR DLPFC) AND (fnirs OR Functional Near-infrared Spectroscopy)) AND (Adult)) AND (Emotions). Filters applied: Clinical Study, Clinical Trial, Randomized Controlled Trial, English, Spanish
Scopus	(TITLE-ABS-KEY (Dorsolateral AND Prefrontal AND Cortex OR DLPFC) AND TITLE-ABS-KEY (fnirs OR nirs OR “Functional Near-infrared Spectroscopy”) AND TITLE-ABS-KEY (adult) AND TITLE-ABS-KEY (emotions)) AND PUBYEAR > 2013 AND PUBYEAR < 2024 AND (LIMIT-TO (DOCTYPE , “ar”)) AND (LIMIT-TO (LANGUAGE , “English”)) AND (LIMIT-TO (SUBJAREA , “MEDI”) OR LIMIT-TO (SUBJAREA , “NEUR”) OR LIMIT-TO (SUBJAREA , “PSYC”))
Web of Science	Dorsolateral Prefrontal Cortex OR DLPFC (Topic) AND fNIRS OR Functional Near-infrared Spectroscopy (Topic) AND Adult (Topic) AND Emotions (Topic). Filters applied: Publication Years: 2013-2024, Article or Clinical Trial. Languages: English. Research Areas: Psychology or Neurosciences Neurology or Psychiatry

### 2.5 Proceso de Selección

**Fig. 1.**
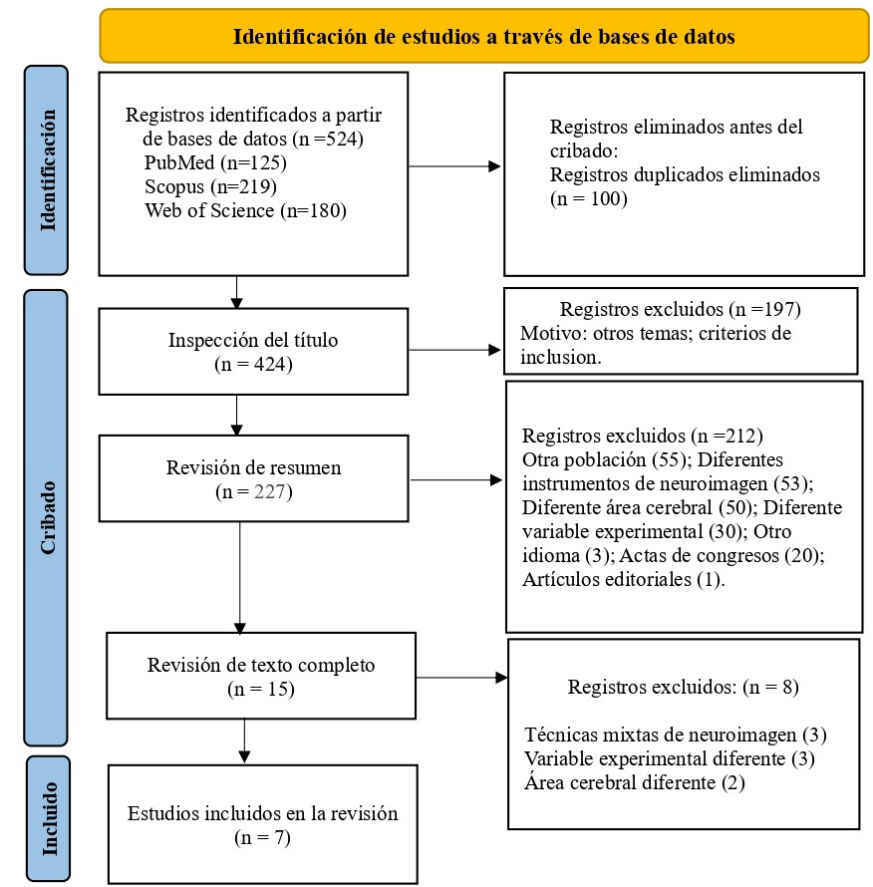
**Diagrama de flujo para revisiones sistemáticas Prisma [21]**.

La revisión sistemática se realizó conforme a los criterios establecidos por la declaración PRISMA [20]. La búsqueda comenzó el 2 de enero de 2024 y finalizó el 21 de enero de 2024. Los artículos fueron seleccionados mediante filtros definidos previamente. Posteriormente, se evaluaron según su relevancia temática y calidad metodológica, mediante el análisis del título, resumen y texto completo. Solo los estudios que cumplían con los criterios de inclusión fueron considerados para el análisis final. Además, se efectuó una búsqueda manual en las listas de referencias de los artículos seleccionados para identificar posibles estudios adicionales (Fig. 1, Ref. [21]).

### 2.6 Proceso de Recolección de Datos 

La extracción de datos se llevó a cabo de forma sistemática. Los registros elegibles se exportaron en formatos CSV y RIS, y se gestionaron mediante Rayyan Bibliographic Manager [22] para la detección de duplicados y clasificación según los criterios de inclusión y exclusión. La selección final de estudios fue realizada por dos revisores independientes (IC y EM), quienes examinaron cada registro en relación con el objetivo de la investigación. Las discrepancias fueron resueltas mediante consenso con un tercer revisor (AR).

### 2.7 Elementos de los Datos

Se recopilaron los siguientes datos: (1) título y autores, (2) revista y cuartil, (3) población y muestra, (4) edad de los participantes, (5) tipo de medición de HbO y HbR, (6) paradigma experimental utilizado para inducir emociones, y (7) región cerebral evaluada mediante fNIRS.

### 2.8 Evaluar el Riesgo de Sesgo del Estudio

La evaluación del riesgo de sesgo se realizó siguiendo las directrices del Joanna Briggs Institute, considerando elementos como la selección de la muestra, las mediciones de HbO y HbR, y la presencia de grupos de comparación. Se aplicó el coeficiente kappa de Cohen para evaluar la concordancia entre los revisores; valores mayores a 0.6 fueron interpretados como acuerdo sustancial, mientras que valores menores a 0.4 indicaron baja concordancia [23].

### 2.9 Análisis de Datos

El análisis de los datos se realizó mediante una sistematización de la información extraída de los estudios seleccionados, organizándolos en una matriz de revisión diseñada ad hoc. Esta matriz incluyó variables clave como autor(es), año de publicación, país de origen, diseño metodológico, población y muestra, objetivos del estudio, técnicas de evaluación utilizadas y principales hallazgos. La sistematización permitió identificar patrones, similitudes y diferencias entre los estudios, así como vacíos teóricos y metodológicos relevantes para el campo de estudio.

## 3. Resultados

Se recuperaron 524 registros de estudios mediante búsquedas en tres bases de datos: PubMed (n = 125), Scopus (n = 219) y Web of Science (n = 180). Tras eliminar 100 registros duplicados, se cribaron 424 publicaciones. De estas, se excluyeron 197 tras la revisión de títulos, y posteriormente 212 más tras el análisis de resúmenes, por no cumplir con los criterios de inclusión. Se evaluaron 15 artículos en texto completo, de los cuales se descartaron 8 por razones metodológicas o temáticas, resultando en un total de 7 estudios incluidos en la revisión sistemática.

Para valorar la calidad metodológica de estos estudios, se utilizó la herramienta del Joanna Briggs Institute [24]. De los 7 estudios, 4 correspondían a diseños transversales y 3 a estudios de casos y controles. La evaluación del riesgo de sesgo fue realizada por dos revisores independientes, y el grado de concordancia entre ellos se estimó mediante el coeficiente *kappa *de* Cohen*. Los resultados detallados de esta evaluación se presentan en la Tabla 3 (Ref. [19, 25, 26, 27, 28, 29, 30]).

**Tabla 3.** **Coeficiente de concordancia Kappa de Cohen**.

Estudios Incluidos	Kappa
Zhang *et al*. (2022) [27]	0,825
Zheng *et al*. (2023) [19]	0,821
Gao *et al*. (2019) [29]	0,778
Dos Santos *et al*. (2022) [26]	1,000
Ernst *et al*. (2013) [25]	0,692
Yeung (2022) [28]	0,692
Sugi *et al*. (2020) [30]	0,692

La concordancia entre los dos revisores en la evaluación del riesgo de sesgo de los siete estudios incluidos varió según el artículo analizado. El estudio de Dos Santos *et al*. [26] presentó el mayor nivel de acuerdo, clasificado como casi perfecto, mientras que el estudio de Zhang *et al*. [27] mostró una concordancia considerable. En contraste, los estudios de Ernst *et al*. [25], Yeung [28] y Sugi *et al*. [30] evidenciaron niveles de concordancia más bajos, ubicándose dentro del rango “moderado”.

Tras el cálculo del coeficiente kappa de Cohen para estimar la consistencia entre evaluadores, se llevó a cabo una revisión conjunta de los resultados. Este proceso permitió alcanzar un consenso unánime respecto a la evaluación del riesgo de sesgo tanto en los estudios con diseño de casos y controles (**Material Suplementario Fig. 1**), como en aquellos con diseño transversal (**Material Suplementario Fig. 2**).

La evaluación del riesgo de sesgo de los estudios de casos y controles determinada en concordancia, posterior a la discusión de las observaciones realizadas por los dos evaluadores, determinó que los tres estudios poseen un criterio no claro Unclear (D2), de la misma manera el estudio de Zheng *et al*. [19] pose un alto riesgo de sesgo en el criterio (D3).

La evaluación del riesgo de sesgo de los estudios transversales determinada en concordancia, posterior a la discusión de las observaciones realizadas por los dos evaluadores, determinó que de los cuatro estudios dos han presentado criterios no claros Unclear, el estudio de Ernst *et al* [25] en los criterios “D7” y “D8”, al igual que el estudio de Yeung (2022) [28] en el criterio “D6” (**Material Suplementario Fig. 2**).

La presente revisión sistemática sintetizó la evidencia disponible sobre la aplicación de fNIRS en el estudio de la actividad de CPFDL durante experimentos emocionales. En primera instancia se revisó los tamaños muestrales de los estudios y estos varían significativamente, Dos Santos *et al*. [26] y Ernst *et al*. [25] presentan muestras relativamente pequeñas, con 33 y 15 participantes, respectivamente. En contraste, el estudio de Zhang *et al*. [27] tiene una muestra considerablemente mayor, con 440 participantes. Otros estudios, como los de Yeung [28] y Sugi *et al*. [30], tienen tamaños de muestra intermedios, con 51 y 30 participantes, respectivamente (véase en la Tabla 4, Ref. [19, 25, 26, 27, 28, 29, 30]).

**Tabla 4.** **Selección de los artículos (n = 7)**.

n	Autor (año)	Revista	Tipo de estudio	Cuartil	Población	Muestra	Media de Edad	DE
1	Dos Santos *et al*. (2022) [26]	*Mindfulness*	Transversal	Q_1_	Adultos sanos	33	33,24	6,85
2	Zhang *et al*. (2022) [27]	*International Journal of Environmental*	Casos y controles	Q_2_	Estudiantes universitarios	440	21,35	3,59
		*Research and Public Health*				HC (220)	HC (21,41)	HC (3,59)
						PD (92)	PD (20,84)	PD (2,83)
						PA (128)	PA (21,58)	PA (4,04)
3	Zheng *et al*. (2023) [19]	*Journal of Affective Disordes*	Casos y controles	Q_1_	Adultos jóvenes	85		
						HC (34)	HC (20,68)	HC (3,73)
						PD with SI (17)	PD with SI (20,06)	PD with SI (2,10)
						PD without SI (34)	PD without SI (20,71)	PD without SI (2,61)
4	Ernst *et al*. (2013) [25]	*CORTEX*	Transversal	Q_2_	Adultos sanos	15	23,4	2,5
5	Yeung (2022) [28]	*Scientific Reports*	Transversal	Q_2_	Adultos sanos	51	25,4	5,6
6	Gao *et al*. (2019) [29]	*Journal of Neural Engineering*	Casos y controles	Q_2_	Adultos	51		
						PD = 27	PD = (40,78)	PD = 27 subject (13,42)
						HC = 24	HC = (43,13)	HC = 24 (11,28)
7	Sugi *et al*. (2020) [30]	*Frontiers in Behavioral Neuroscience*	Transversal	Q_3_	Adultos sanos	30	21,6	0,9

*Nota.* HC, Control Sano; PD, Grupo de depresión; PA, Grupo de ansiedad; PD with SI, Pacientes con ideación suicida; PD without SI, Pacientes sin ideación suicida; DE, Desviación estánda.

La edad promedio (20–45 años) y la desviación estándar varían entre los estudios, reflejando diferencias en las características de las muestras. La calidad de las revistas en las que se publicaron estos estudios es en revistas de alto impacto Q_1_ (29%) y otros en revistas de impacto Q_2_ (57%) y Q_3_ (14%), esto refleja un equilibrio en la calidad y el alcance de las revistas donde se publicaron los estudios.

Los trabajos están distribuidos en siete revistas diferentes, *CORTEX*, *Frontiers in Behavioral Neuroscience*, *International Journal of Environmental Research and Public Health*, *Journal of Affective Disorders*, *Journal of Neural Engineering*, *Mindfulness* y *Scientific Reports*. Esta diversidad de fuentes indica que la investigación está siendo abordada desde múltiples perspectivas académicas y científicas, asimismo, que están creciendo en los últimos tiempos (**Material Suplementario Tabla 1**). Además, la distribución por continente muestra que la mayor parte de la investigación proviene de Asia, con cinco artículos (71%). América y Europa están representadas cada una con un artículo (14%).

Consecuentemente se analizaron los estudios en cuanto a la metodología, presentación de resultados y aportes. Hay investigaciones novedosas que involucran otros aspectos como mindfulness, en los que investigan Dos Santos *et al*. [26] sobre los efectos del pensamiento autocompasivo (SCT), relacionado con recuerdos autobiográficos estresantes (SAM) sobre la actividad de la CPF y los parámetros de variabilidad de la frecuencia cardíaca (VFC), en sujetos sanos (n = 33 hombres). Se utilizó un NIRSport system de 20 canales, el experimento con un diseño de bloques que consistía en escuchar audios con las dos condiciones con 45 segundos de exposición y 25 segundos de control; lo que se observó fue que en la condición SAM, la CPFDL izquierda y el área frontopolar (área 10 de Brodmann) mostraron una concentración de oxihemoglobina significativamente mayor en comparación con la condición de control. Durante la condición SCT, el área frontopolar mostró un aumento significativo de oxihemoglobina en comparación con la condición de control [26]. Si bien hay una una asociación entre la señal fNIRS del área frontopolar y el *high-frequency component of heart rate variability* (HF-HRV) durante la condición SAM, la relación con los recuerdos autobiográficos estresantes está asociada con actividad en el CPFDL izquierdo y el área frontopolar, mostrando un aumento de activación en las zonas indicadas; mientras que el pensamiento autocompasivo es una forma adaptativa de pensar acerca de situaciones estresantes y esto puede considerarse una especie de enfoque de reevaluación que presenta una actividad en el área frontopolar; por lo que puede ser una estrategia eficaz de regulación emocional [26].

Zhang *et al*. [27], investigan las características del fNIRS en CPFDL en estudiantes universitarios ansiosos (n = 128) y deprimidos (n = 92) en relación con un grupo control (n = 220) con una tarea de memoria autobiográfica emocional (EAMT). Utilizó un fNIRS de 53 canales, y realizó una tarea con un diseño de bloques en la que se presentó las imágenes Sistema Chino de Imágenes afectivas(CAPS) [31], y se solicitó a que el participante recuerde eventos que han experimentado con cada imagen. Se inicia la tarea con un descanso de 30 segundos, posterior una imagen (positiva, neutra o negativa) con 60 segundos de exposición y un descanso de 30 segundos entre cada imagen. Los resultados señalan que en el grupo de depresión (ch13), la activación del CPFDL izquierdo fue notablemente superior a la del grupo de ansiedad. La activación del CPFDL derecho (ch 48-ch 41) mostró una relación significativamente mayor con emociones positivas en comparación con las negativas, en el grupo de depresión; mientras que, bajo emociones negativas, el grupo de depresión experimentó una activación inferior a la del grupo sano. En estudiantes con síntomas de alta ansiedad, el CPFDL derecho mostró una activación reducida frente a las emociones positivas, así como una activación reducida frente a las emociones negativas en aquellos con depresión. Además, a mayor nivel de ansiedad, menor es la activación en el lado izquierdo y mayor es la activación en el lado derecho, lo que indica que la ansiedad podría disminuir la función del CPFDL izquierdo. [27].

Del mismo modo, Zheng *et al*. [19], evaluaron la actividad cerebral en CPFDL en personas deprimidas con (n = 17) y sin ideación suicida (n = 34) y se comparó con un grupo control (n = 34). La oxihemoglobina promedio en CPFDL de los sujetos durante EAMT se recopiló mediante un dispositivo de imágenes fNIRS de 53 canales, que incluyó aspectos específicos del pasado e información relacionada a la persona que se activa mediante imágenes previamente seleccionadas del sistema CAPS, en la cual se le pide al participante que recuerde los eventos asociados a cada imagen. Se inicia con un descanso de 30 segundos, posterior una imagen (positiva, neutra o negativa) con 60 segundos de exposición y un descanso de 30 segundos entre cada imagen. Se observó que, en presencia de la emoción negativa, el grupo con depresión sin ideación suicida tuvo una activación superior que el grupo control en la CPFDL izquierda; mientras que, bajo la emoción positiva, el grupo de depresión con ideación suicida presentó una activación inferior que el control sano en la CPFDL derecha, lo que sugiere alguna disfuncionalidad en esa zona cortical. Según los autores los pacientes con ideación suicida tenían algunos déficit en la función ejecutiva en la CPFDL derecha, mientras que los adultos deprimidos sin ideación suicida pueden tener un mecanismo de reclutamiento compensatorio de recursos en CPFDL izquierdo [19].

Por otra parte el estudio de Ernst *et al*. [25], midió la actividad de la CPF mediante fNIRS de 52 canales durante la ejecución una tarea de evitación y acercamiento (AAT); una versión con joystick, en el experimento, los participantes (n = 15) acercaron (hacia su cuerpo) y evitaron (alejado de su cuerpo) imágenes positivas y negativas International Affective Picture System (IAPS) [32], que se presentaron 3–8 segundos con 5 segundos en el intervalo con una cruz de fijación. Las reacciones reguladas incompatibles (evitar lo positivo, acercarse a lo negativo) en comparación con las reacciones automáticas compatibles (aproximarse a lo positivo, evitar lo negativo) causaron una activación más fuerte en la CPFDL derecha.

Asimismo, en el mismo estudio, en el contexto de las tendencias de abordaje en los trastornos de adicción, se presentó imágenes con y sin alcohol en el experimento, en el cual la corteza orbitofrontal lateral anterior izquierda como parte del sistema de recompensa general que procesa las recompensas secundarias, mostró una activación más fuerte en términos de aumento de OHb durante el acercamiento en comparación con evitar cuadros de alcohol. Esta diferencia se correlacionó positivamente con las expectativas de los participantes sobre los efectos beneficiosos del alcohol en términos de regulación emocional [25]

Por otro lado, en la investigación de Yeung [28] en estudiantes universitarios (n = 51), se llevó a cabo una prueba de fluidez semántica (SFT) en comparación con la prueba verbal convencional mediante un fNIRS de 48 canales que tuvo un diseño con un tiempo de inicio de 30 segundos con 60 segundos por cada bloque, donde cada participante debió describir la mayor cantidad de palabras según las categorías de, palabras sin emoción (país, ocupación) y de emoción (positiva, negativa). El estudio reporta que hubo aumentos significativos en la concentración de HbO en las regiones frontopolar, dorsolateral y ventrolateral frontal durante la prueba verbal emocional y no emocional, concluyendo que la activación cortical frontal durante la prueba de fluidez semántica y convencional es similar [28].

En el estudio de Gao *et al*. [29] sobre el reconocimiento de emociones faciales en personas con depresión (n = 27) y controles sanos (n = 24), se utilizó un fNIRS de 6 canales, la tarea consistió en observar imágenes de caras emocionales en escala de grises de felicidad, temor y tristeza (40 pruebas), y la presentación duraba un segundo, intercalada con un asterisco de 3 segundos de duración, el participante tenía que reconocer cuales son las imágenes congruentes y cuales son incongruentes. Los principales resultados fueron los cambios en la CPF izquierda que tuvo diferencias significativas en relación con los pacientes con depresión, siendo la HbO, más baja que en personas sanas [29].

Mientras que, Sugi *et al*. [30] estudiaron la personalidad, en dos categorías; el grupo del sistema de inhibición del comportamiento (BIS) y el grupo del sistema de activación del comportamiento (BAS), según sus puntuaciones en la escala BIS/BAS de rasgos de personalidad. Los participantes (n = 30) realizaron una tarea de memoria con estímulos auditivos, verbales y visoespaciales que contemplaba observar seis imágenes emocionales (negativa, neutra y positiva) (duración de 1 segundo por cada una), que fueron seleccionadas del IAPS, en el cual se utilizó un fNIRS de 44 canales y se midió la concentración de cambios de HbO en el CPFDL durante la tarea. Los resultados señalan un tiempo de reacción (RT) significativamente mayor en la valencia negativa independientemente de los rasgos de personalidad. Además, la actividad de la CPFDL fue significativamente mayor en estímulos negativos que en la valencia neutra en el grupo BIS; cabe indicar que la actividad de la CPFDL derecha también fue significativamente mayor en el grupo BIS que en el grupo BAS en la valencia positiva, lo que podría indicar un mayor control de la emoción [33]. No hubo ningún efecto principal o interacción en la actividad de la CPFDL izquierda [30].

## 4. Discusión

Las mediciones de la actividad cerebral mediante fNIRS analizadas, muestra una variedad de equipos (China, Japón, Alemania) y configuraciones técnicas utilizadas en los diferentes estudios. Estos incluyen sistemas con diferentes números de canales, longitudes de onda de luz y tasas de muestreo, lo que resalta la importancia de adaptar la tecnología a las necesidades específicas de cada investigación.

Los experimentos relacionados con la evaluación de las emociones involucran diversas tareas, cada una diseñada para investigar aspectos específicos de la regulación emocional y la neurocognición; como la tarea de memoria autobiográfica emocional (EAMT), tarea de recuerdos autobiográficos estresantes (SAM), pensamiento autocompasivo (mindfulness) (STC), tarea de memoria autobiográfica autoemocional (EAMT), tarea de evitación y acercamiento (AAT), el test emocional analógico de la Fluidez Semántica (SFT) tarea de reconocimiento facial, entre otros. Estos experimentos proporcionan una visión de cómo los individuos procesan y responden a diferentes estímulos emocionales y la versatilidad de tareas que se pueden realizar para medir aspectos emocionales.

Por otra parte, el conjunto de instrumentos utilizados en los diferentes estudios consta de una variedad de herramientas de evaluación, incluyendo el Cuestionario de Lateralidad de Edimburgo, el Inventario de Ansiedad Estado-Rasgo, el Inventario de Depresión de Beck (BDI), la Escala BIS/BAS, la versión japonesa. Además, se emplearon pruebas como, el DASS-21, el HADS, la Escala de Valoración de la Depresión, la Escala de Idea de Suicidio de SIOSS, Mini Entrevista Neuropsiquiátrica Internacional (MINI-versión china), Positive Affect Negative Affect Schedule (PANAS), State-Trait-Anxiety-Inventory (STAI-X2), Cuestionario Breve de Expectativas de Alcohol (Brief AEQ-G) y el Hamilton Rating Scale para la Depresión (HRSD), entre otros.

Consecuentemente los resultados de la activación cerebral indican patrones distintivos en diferentes regiones cerebrales, se reporta en todos los estudios las mediciones de HbO-HbR, las zonas que se activan según el tipo de estímulo emocional, en la presencia de trastornos de ansiedad o depresión, e incluso el consumo de alcohol, se observa una alteración en la CPFDL izquierdo. En relación con adultos sanos se observa una activación en la CPFDL ante las tareas realizadas, que refleja el proceso neurocognitivo subyacente de selección, análisis y toma de decisión entre otros. Estos hallazgos destacan la complejidad de los mecanismos subyacentes a la regulación emocional y la importancia de tener en cuenta una variedad de factores en la investigación en este campo.

En síntesis, estos resultados proporcionaron una visión detallada y completa de los hallazgos de la investigación en neurociencia cognitiva y emocional utilizando fNIRS, además revelaron la diversidad de enfoques metodológicos, la importancia de considerar una variedad de factores contextuales y la complejidad de los procesos subyacentes a la neurocognición y la emoción. Estos hallazgos tienen implicaciones importantes tanto para la teoría como para la práctica en campos como la psicología, la neurociencia y la salud mental (Tabla 5, Ref. [19, 25, 26, 27, 28, 29, 30]).

**Tabla 5.** **Características de los estudios de fNIRS (n = 7)**.

n	Autor (Año)	fNIRS	Experimento emocional	HbO (Grupo control > Pacientes)	HbR (Grupo control > Pacientes)
1	Dos Santos *et al*. (2022) [26]	NIRSport (NIRx Medical Technologies, Alemania), 20 canales	Diseño en bloques; dos condiciones SAM. SCT. Audios de 45 s, control de 25 s, repetido 5 veces.	SAM, corteza prefrontal izquierda (canales 3, 5, 6); SCT, corteza frontal (canales 9, 12)	SAM, corteza frontal (canal 12); SCT, corteza prefrontal izquierda (canal 2)
2	Zhang *et al*. (2022) [27]	fNIRS (BS-7000, Wuhan Znion Technology Co., China), 53 canales	Diseño en bloques: Tarea de memoria autobiográfica emocional (EAMT). 30 s de descanso, 60 s de imágenes emocionales (positivas, negativas, neutras), repetido 3 veces.	Mayor activación en CPFDL izquierdo en DP frente a TA; DP > activación con emociones negativas	TA < activación con emociones positivas
3	Zheng *et al*. (2023) [19]	fNIRS (BS-7000, Wuhan Znion Technology Co., China), 53 canales	Diseño en bloques: Tarea de memoria autobiográfica emocional (EAMT). 30 s descanso, 60 s imágenes emocionales (positivas, negativas, neutras), repetido 3 veces.	DP sin IS > activación en CPFDL izquierdo (emoción negativa)	DP con IS < activación en CPFDL derecho (emoción positiva)
4	Ernst *et al*. (2013) [25]	fNIRS (ETG-4000, Hitachi Medical Co., Japón), 52 canales	Versión con joystick de la tarea de aproximación-evitación (AAT). 60 ensayos (10 imágenes, 2 categorías: positiva-negativa, 3 repeticiones).	Corteza prefrontal frontal (canales 6, 15, 16, 27) > activación en emociones positivas	Menor activación en CPFDL derecho
5	Yeung (2022) [28]	fNIRS (ETG-4000, Hitachi Medical Co., Japón), 48 canales	Tarea de fluidez semántica emocional (SFT). 30 s de inicio, 60 s de tarea SFT.	SFT no emocional: aumento de activación frontal izquierda; activación bilateral frente a emociones	SFT no emocional: menor activación en corteza prefrontal dorsomedial y frontal. Sin diferencias significativas
6	Gao *et al*. (2019) [29]	fNIRS (CW-NIRS, Universidad Jiaotong de Xi’an, China), 6 canales	Reconocimiento emocional facial (alegría, miedo, tristeza). 40 ensayos por emoción (20 congruentes, 20 incongruentes).	DP menor actividad en corteza prefrontal izquierda que SC	Activación menor en corteza prefrontal izquierda en DP
7	Sugi *et al*. (2020) [30]	fNIRS (LABNIRS, Shimadzu Corporation, Kioto, Japón), 44 canales	Tarea de memoria de trabajo N-back (auditiva-verbal y visuoespacial) con estímulos emocionales (negativos, neutros, positivos). 6 bloques (16 estímulos por bloque).	Mayor activación en CPFDL derecho con emociones negativas frente a neutras	Sin diferencias significativas

*Nota*: SAM, Stressful autobiographichal memories/ Memoria autobiográfica estresante; SCT, Self compassionate thinking/ Pensamiento autocompasivo; CPFDL, corteza prefrontal dorsolateral; CPF, Corteza prefrontal; HC, Control Sano; DP, Grupo de depresión; PA, grupo de ansiedad; DP with IS, Paciente con depresión con ideación suicida; DP without IS, Paciente con depresión sin ideación suicida; BIS, sistema de inhibición del comportamiento; BAS, sistema de activación del comportamiento; > mayor que, < menor que.

Los resultados obtenidos ofrecen una visión integral de cómo esta técnica no invasiva ha contribuido a evaluar los mecanismos neurales subyacentes a los aspectos emocionales en la CPFDL, ya que esta es una región cerebral crucial para las capacidades neurocognitivas más sofisticadas, ya que permite planificar, organizar, recordar, razonar, tomar decisiones, controlar nuestras emociones y adaptarnos a un mundo cambiante [18, 34]. La revisión sistemática evidencia que la activación de la CPFDL, medida mediante fNIRS, varía según el tipo de estímulo emocional y la condición clínica. Se observó hipoactivación en la CPFDL izquierda en personas con depresión y ansiedad, y mayor activación en la CPFDL derecha ante estímulos positivos. Estos hallazgos refuerzan el potencial del fNIRS como herramienta útil en la evaluación emocional y la investigación sobre la lateralidad cerebral.

La activación de esta zona cerebral se registró de manera efectiva mediante el fNIRS en las tareas que involucran un procesamiento emocional; además demostró una alteración o hipoactivación de CPFDL izquierda lo que puede ser indicador de patología en casos de depresión, ansiedad, ideación suicida, en comparación a los controles sanos; lo cual respalda las intervenciones por ejemplo con neuromodulación no invasiva como tratamiento para estos trastornos mentales [35]. Se observa que la CPFDL derecha se activa ante estímulos agradables y en actividades de autocompasión o reestructuración cognitiva lo cual abre una puerta para futuras intervenciones e investigación sobre lateralidad cerebral. Asimismo, la revisión destaca el creciente rigor metodológico de los estudios con fNIRS, lo que contribuye a fortalecer la base empírica de la psicología, neurociencias, entre otras [6].

Se observó diferentes sistemas y configuraciones de canales para la detección de cambios hemodinámicos en áreas cerebrales específicas; además del número de receptores ópticos que se utilizan, algunos de 53 otros de 6 canales y los sistemas de medición de ondas continuas, con distintas longitudes de onda y tasas de muestreo [15, 27, 28, 30, 36].

Los experimentos desarrollados en los estudios incluidos en la revisión abordaron una variedad de procesos neurocognitivos y emocionales, como los recuerdos estresantes, la percepción emocional, la ansiedad, la depresión y el estrés, utilizando tareas conductuales y estímulos emocionales para inducir respuestas específicas. Se utilizaron tareas de memoria autobiográfica emocional, tareas de n-back dual, y tareas de reconocimiento de emociones faciales, utilizando imágenes estandarizadas como es el IAPS, CAPS, base de datos de rostros Extended-Cohn-Kanade, demostrando la versatilidad de las investigaciones que se pueden realizar con este dispositivo y la creciente investigación que se está realizando en este ámbito. Estos hallazgos sugirieron que fNIRS puede ser una herramienta útil para diagnosticar y caracterizar a pacientes sanos y con trastornos del estado de ánimo.

Los datos obtenidos incluyeron mediciones de HbO y HbR, así como de la hemoglobina total (Total-Hb), utilizando los principios de la ley de Beer-Lambert para calcular cambios en la oxigenación cerebral. Se llevaron a cabo análisis estadísticos para evaluar la actividad cerebral en respuesta a diferentes estímulos y condiciones experimentales, identificando regiones activas y asociaciones entre variables fisiológicas y psicológicas, lo que se ha identificado a través de esta revisión es que hay una diversidad de análisis estadísticos, lo cual es una limitación considerable ya que no se reportan los resultados de la misma manera lo que puede restringir la exploración adecuada de los datos, la unificación de resultados y metaanálisis posteriores [12, 37].

Los hallazgos de esta revisión sugieren aspectos teóricos, metodológicos y prácticos, entre los que describen que para mejorar la calidad de los datos obtenidos mediante fNIRS, es necesario optimizar los parámetros experimentales. En los estudios de Ernst *et al*. [25], Yeung [28], Gao *et al*. [29], Sugi *et al*. [30] se observó un tiempo de exposición ante las imágenes de entre 1–2 segundos lo cual según otros investigadores [37, 38] se recomienda emplear diseños por bloques y que los tiempos de exposición a los estímulos de entre 5 a 10 segundos y un tiempo de intervalo de entre 8–15 segundos, lo que permitirá obtener señales más claras, precisas, y que facilite la interpretación de los resultados [2].

Las fortalezas de esta técnica de neuroimagen son que es una herramienta complementaria a la RMf o EEG y, especialmente útil en poblaciones que no pueden acceder a estas técnicas. Su portabilidad y facilidad de uso permiten su implementación en entornos clínicos, facilitando la evaluación de pacientes con diversas condiciones neurológicas y psicopatológicas. Además, su capacidad para medir la actividad cerebral en tiempo real abre nuevas posibilidades para la investigación en neurociencia clínica. Así, la literatura revisada no solo destaca el crecimiento y la evolución de fNIRS como herramienta de investigación, sino que también subraya su versatilidad y relevancia en diversas áreas de la neurociencia, lo que abre nuevas oportunidades para la investigación y la aplicación clínica.

Dentro de las posibles limitaciones observadas son los tamaños muéstrales de las investigaciones, y las características metodológicas propias de la recepción de la señal, la posibilidad de un análisis adecuado de los datos, mediante los filtros correspondientes y la presentación de resultados que no se reportan de manera similar en los estudios [3, 12]. Si bien la revisión proporciona una visión general de los estudios, la variedad de tareas y usos para el fNIRS utilizados en los experimentos puede dificultar la comparación directa de los resultados, así como también la heterogeneidad en los diseños experimentales y las muestras podría reflejar una limitación en la generalización de resultados.

Se sugiere para futuras investigaciones ampliar la revisión sistemática a diferentes tipos de neuroimagen que podría proporcionar información valiosa sobre los beneficios otras intervenciones relacionadas a la medición de la CPFDL y la emoción. Es fundamental que los estudios futuros incluyan una variedad de grupos demográficos y clínicos, como personas con trastornos emocionales o enfermedades mentales, para mejorar la generalización de los resultados. Con el objetivo de comprender los efectos a largo plazo de las emociones en la actividad cerebral reportados por otros estudios, se recomienda realizar revisiones que involucren únicamente estudios longitudinales que estimen a los participantes a lo largo del tiempo de investigación.

## 5. Conclusión

En conclusión, los hallazgos reportados por la presente revisión tienen un impacto significativo en la comprensión de la regulación emocional en individuos sanos y clínicos, ayudando a desarrollar modelos más precisos para las emociones y la neurocognición, además de tener aplicaciones importantes en el diagnóstico y tratamiento de trastornos emocionales, describiendo las propiedades de una herramienta no invasiva para evaluar la actividad cerebral en diferentes contextos. Además se amplía una gran posibilidad de estudios relacionados a personas sanas, con psicopatologías, trastornos neurocognitivos entre otros.

## Contribuciones de los Autores

Contribución a la concepción y el diseño: ICT; Contribución a la recopilación de datos: ICT, EAMM, GBS, AR y JDY; Contribución al análisis y la interpretación de datos: ICT, GBS, AR y JDY; Redacción y revisión del artículo: ICT, EAMM, GBS, AR y JDY. Todos los autores aprobaron el manuscrito final. Todos los autores participaron adecuadamente en el trabajo y aceptaron ser responsables de todos sus aspectos.

## Aprobación Ética y Consentimiento Informado

No aplicable.

## Agradecimientos

Los autores agradecen al Instituto de Neurociencias de la Universidad Católica de Cuenca y la Universidad Autónoma de Barcelona.

## Financiación

Este trabajo fue apoyado por la Universidad Católica de Cuenca, con el proyecto de investigación PICODS21-20.

## Material Suplementario

Así como también el **Material Suplementario–Video** del corresponde a un resumen del estudio. El material suplementario asociado con este artículo se puede encontrar, en la versión en línea, en https://doi.org/10.31083/RN44275.
