# Supplementary material for: fNIRS in Emotional Appraisal and the Dorsolateral Prefrontal Cortex: A Systematic Review
Source: Rev Neurol. 2025 Nov 30;80(10):44275. [Article in Spanish] doi: 10.31083/RN44275 (PMC12680975; doi:10.31083/RN44275)
Supplement: Supplementary file 1 [file 1576-6578-80-10-44275-s1.zip › Material Suplementario.docx]

**Anexos**

***Tabla Complementaria 1.*** *Análisis descriptivo de los artículos seleccionados.*

|  | **n (%)** |
| --- | --- |
| **Año** |  |
| 2011 | 1 (14%) |
| 2019 | 1 (14%) |
| 2020 | 1 (14%) |
| 2021 | 1 (14%) |
| 2022 | 2 (29%) |
| 2023 | 1 (14%) |
| **Revista** |  |
| CORTEX | 1 (14%) |
| Frontiers in Behavioral Neuroscience | 1 (14%) |
| International Journal of Environmental Research and Public Health | 1 (14%) |
| Journal of Affective Disordes | 1 (14%) |
| Journal of Neural Engineering | 1 (14%) |
| Mindfulness | 1 (14%) |
| Scientific Reports | 1 (14%) |
| **Cuartil** |  |
| Q1 | 2 (29%) |
| Q2 | 4 (57%) |
| Q3 | 1 (14%) |
| **País** |  |
| China | 4 (57%) |
| Germany | 1 (14%) |
| Brasil | 1 (14%) |
| Japón | 1 (14%) |
| **Continente** |  |
| América | 1 (14%) |
| Asia | 5 (71%) |
| Europa | 1 (14%) |

**Figuras complementarias**

| **Figura 1.** *Evaluación del riesgo de sesgo de acuerdo a los criterios del Instituto Joanna Briggs para estudios con diseños de casos y controles.* |
| --- |
| 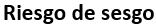*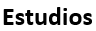*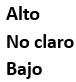**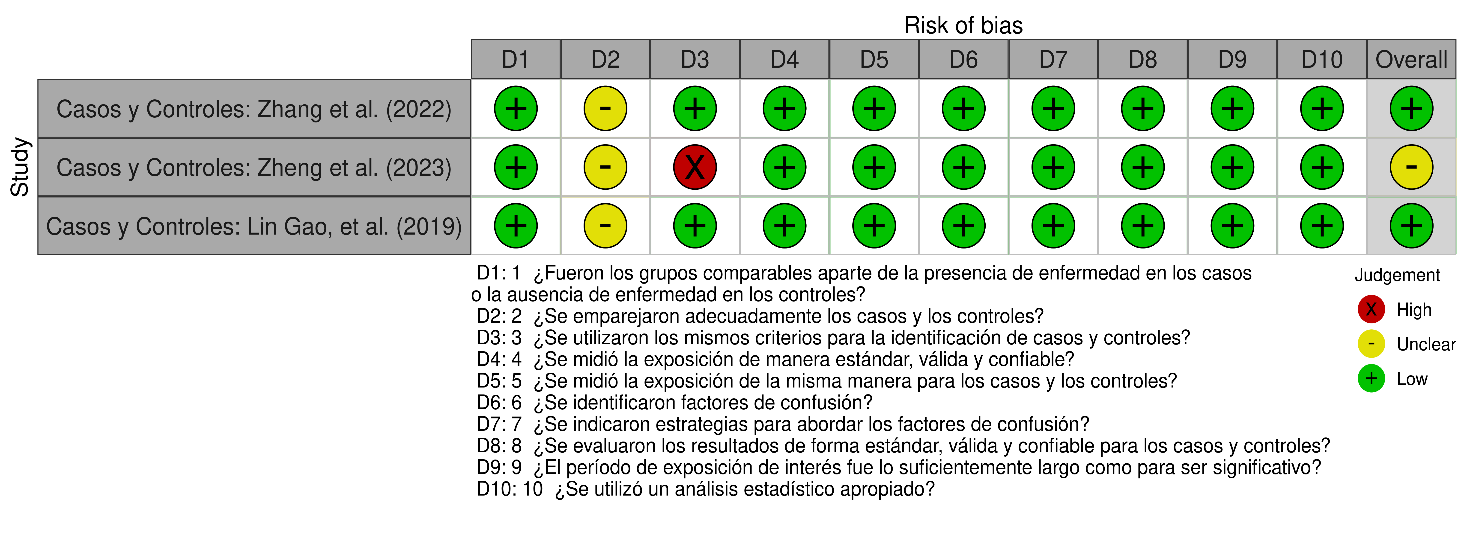** |

| **Figura 2.** *Evaluación del riesgo de sesgo de acuerdo a los criterios del Instituto Joanna Briggs para estudios con diseños transversales.* |
| --- |
| 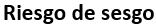*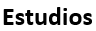*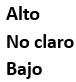**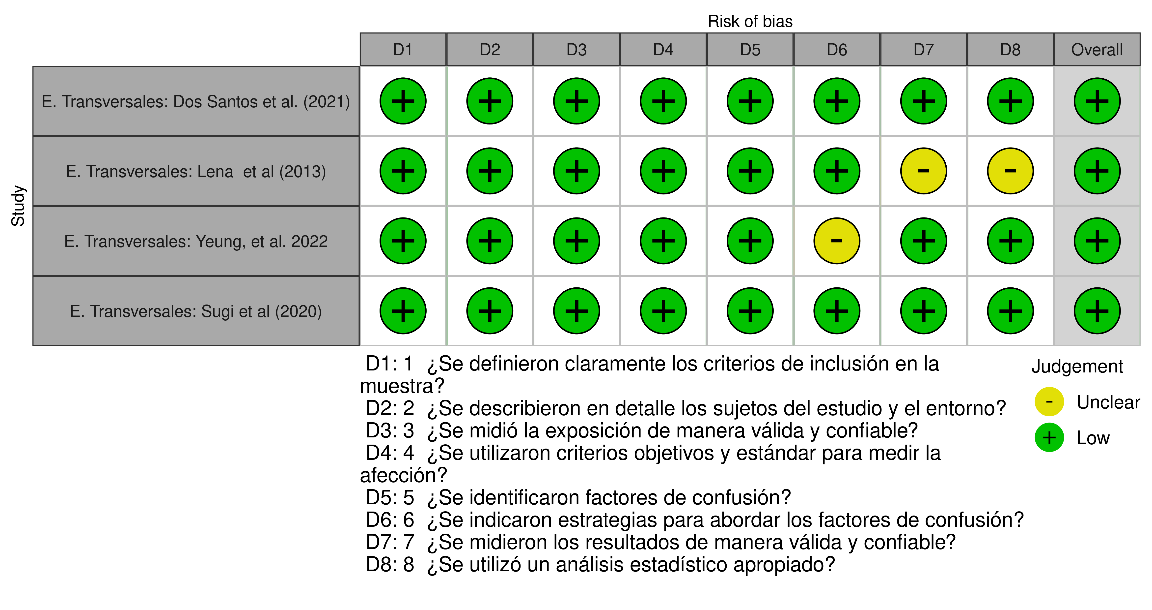** |

Glosario

| CPFDL | Corteza Prefrontal Dorsolateral |
| --- | --- |
| fNIRS | Espectroscopia Funcional de Infrarrojo Cercano |
| NIRS | Espectroscopia infrarrojo cercano |
| RMf | Resonancia Magnética |
| HbO | Oxihemoglobina |
| HbR | Desoxihemoglobina |
| EEG | Electroencefalograma |
| RT | Tiempo de reacción |
| VFC | Variabilidad de la frecuencia cardíaca |
| HC | Helthy control |
| PD | Depressión group |
| PA | Anxiety group |
| PDwith SI | Patient with suicidal ideation |
| PDwithoutSI | Patient without suicidal ideation |
| PFC | Prefrontal cortex |
| CPF | Corteza prefrontal |
